# The Elements of Bandaging and the Treatment of Fractures and Dislocations

**Published:** 1914-03

**Authors:** 


**The Elements of Bandaging and the Treatment of Fractures and Dislocations.** By WILLIAM RANKIN, M.A., M.B., Ch.B. Pp. x. 116. London: Oxford Medical Publications. 1913. Price 5s. net.

As stated in the Introduction, this manual deals essentially with details which are not easily found in most text-books. It is intended for practitioners of limited experience. The commonest conditions are those chiefly described, and the details of treatment given are those which have proved most satisfactory in practice.

The writer forcibly advocates the use of anæsthesia in the diagnosis and treatment of fractures and dislocations, and is of the opinion that without it, in most cases, "the examination will be incomplete and of necessity the treatment will be defective." He writes, "An anæsthetic is routine practice in every properly conducted surgical clinique whenever required, and ought to be so in general practice"; and again, under dislocations which "of course can be reduced without anæsthetics; any brute of sufficient physique who has a little knowledge can manage it; the question is whether he would permit it to be carried out on his own person, or in the case of one of his near relatives."

In our opinion this small manual is a good one. It is sufficiently illustrated, and gives in the text many serviceable details of the treatment of fractures which we have not seen noticed elsewhere.

